# Task modulation of disyllabic spoken word recognition in Mandarin Chinese: a unimodal ERP study

**DOI:** 10.1038/srep25916

**Published:** 2016-05-16

**Authors:** Xianjun Huang, Jin-Chen Yang, Ruohan Chang, Chunyan Guo

**Affiliations:** 1 Beijing Key Laboratory of Learning and Cognition and Department of Psychology, Capital Normal University, Beijing, China; 2 Center for Mind and Brain, University of California Davis, Davis, CA, USA; 3 Department of Neurology, University of California Davis, School of Medicine, Sacramento, CA, USA; 4 Institute of Psychology, Chinese Academy of Sciences, Beijing, China

## Abstract

Using unimodal auditory tasks of word-matching and meaning-matching, this study investigated how the phonological and semantic processes in Chinese disyllabic spoken word recognition are modulated by top-down mechanism induced by experimental tasks. Both semantic similarity and word-initial phonological similarity between the primes and targets were manipulated. Results showed that at early stage of recognition (~150–250 ms), an enhanced P2 was elicited by the word-initial phonological mismatch in both tasks. In ~300–500 ms, a fronto-central negative component was elicited by word-initial phonological similarities in the word-matching task, while a parietal negativity was elicited by semantically unrelated primes in the meaning-matching task, indicating that both the semantic and phonological processes can be involved in this time window, depending on the task requirements. In the late stage (~500–700 ms), a centro-parietal Late N400 was elicited in both tasks, but with a larger effect in the meaning-matching task than in the word-matching task. This finding suggests that the semantic representation of the spoken words can be activated automatically in the late stage of recognition, even when semantic processing is not required. However, the magnitude of the semantic activation is modulated by task requirements.

One of the characteristic features of the speech signal is its dynamic and temporal nature - speech signal unfolds over time and is transient. To accomplish a correct recognition, listeners have to extract the right abstract representations, including phonological and semantic ones, from the rapidly changing acoustic signal of the running speech stream, and map them onto words in the mental lexicon. The term “lexical access” usually describes the moment at which all lexical information becomes available to the speech processor^1^. There is a large body of evidence indicating that spoken word access is modulated by the phonetic and semantic details of speech at pre-lexical and lexical levels respectively^2^,^3^,^4^,^5^,^6^.

As demonstrated by studies using the gating paradigms, most words can be identified before their acoustic offset^7^. Event-related brain potentials (ERP) offer an excellent noninvasive tool for measuring the time course of ongoing neural events at both pre-lexical and lexical levels during spoken word recognition, with exquisite temporal resolution (millisecond level). There are several ERP components known to be associated with the phonological and semantic processes; namely, the Phonological mismatch negativity (PMN), the P2, and the N400.

The phonological processing at early stages of word recognition can be reflected by the PMN and P2 effects. An early negative component appearing about 200–250 ms post-stimulus onset can be elicited by word-initial phonological mismatches during terminal word recognition in spoken sentences^2^, as well as in picture-spoken word matching tasks^8^,^9^. Though considered by some researchers^5^,^6^ as the early portion of the N400 component reflecting lexical selection processes, this negative component is more generally viewed as the PMN, an ERP that reflects the acoustic-phonetic processing of the initial phoneme of a spoken word.

It has been well-established that the N400 component is a family of components associated with multi-dimensional information in language processing. The classic N400 can index semantic processing and/or semantic integration^10^,^11^,^12^. The N400 has also been demonstrated to be sensitive to phonological processing during spoken word access. In phonological priming studies, when the rhyme of the target is different from that of the prime, a negative N400-like component can be elicited. This phenomenon is known as the phonological N400 or the rhyming effects^3^,^4^.

The phonological and semantic processes indexed by the N400 effect, were found to be modulated by top-down mechanisms in several studies^13^,^14^,^15^. The phonological N400 effect only appeared in the active task, but the semantic N400 effect existed in both the active and passive tasks^13^. The semantic N400 was significantly larger and lasted longer than the phonological N400, indicating that the effect of the semantic incongruence is more robust and automatic than the effect of the phonological incongruence^13^. Yoncheva and colleagues examined whether the auditory ERP effects of rhyming were automatically elicited by spoken words or reflect an active process denpendant on the listeners’ attention to the phonology by using a selective attention paradigm^14^,^15^. They found that the rhyming similarity effects emerged only when attention was directed to phonology (i.e., in the rhyming judgment task) but were not evident in the tone-triplet matching task. These results demonstrated that top-down mechanisms such as task requirement and attention allocation can modulate spoken word processing, especially the phonological processing.

However, the phonological N400 effect found in prior studies were examined by manipulating the word-final phonological similarity (i.e., with similar or dissimilar rhymes between the primes and the targets). Temporal and spatial overlaps exist between the phonological N400 effect and the semantic N400 effect, which may confound the experimental effects obtained.

Most of the studies on spoken word recognition are based on Indo-European languages, with relatively few studies on tonal languages such as Mandarin Chinese. Studying Chinese spoken word recognition thus can offer opportunity to examine the language-universal and language-specific aspects of the top-down modulation during spoken word access. Furthermore, researchers have suggested that a cross-modal task may not activate the most typically used spoken word processing routes^16^. Huang and colleagues investigated the time course of spoken word recognition in Mandarin Chinese using a unimodal word-matching paradigm, in which the prime and target words were both presented auditorily^17^. Disyllabic words were used as stimuli in their experiment 1 and the phonological relations between the words in pairs were manipulated, with the same target word preceded by identical, unrelated, or cohort primes (i.e., sharing the same initial syllable as the target). They found an enhancement of an anteriorly distributed P2 component between 200 and 270 ms induced by word-initial phonological mismatches. Monosyllabic words were used as stimuli in their experiment 2, in which semantic relation between the primes and targets was manipulated: besides the identical and unrelated primes, phonologically-unrelated antonymous primes were used to examine whether the phonological mismatch effect was associated with semantic activation. A P2 effect as in experiment 1 was replicated in experiment 2, and the phonological mismatch-associated P2 did not appear to be modulated by semantic processing, suggesting that there is an early phonological processing stage independent of semantic processing in spoken word recognition.

In the current study, both the word-initial phonological similarities and the semantic similarities between the prime and target words were manipulated to investigate how the phonological processing and semantic processing during spoken word recognition in Mandarin Chinese are modulated by top-down mechanisms introduced by task requirements. Two unimodal (auditory) tasks were utilized, one of which was also used by Huang and colleagues^17^, i.e., the word-matching task (Task 1). In this task, the participants were instructed to decide if the prime and the target word were the same. This task is more phonologically oriented, because a judgment can be made without using the meanings of the words. Therefore, in addition to the word-matching task, we also utilized a meaning-matching task, in which the participants were asked to decide whether the meanings of the words in each pair were the same or not (Task 2). Task modulation on the time course of phonological and semantic processing was examined by comparing the ERPs induced by the two tasks.

## Results

### Behavioral results of Group 1 stimuli in Task 1 and Task 2

Reaction time (RT) and accuracy data for the Group 1 stimuli in both tasks are presented in Table 1.

Two-way repeated measures ANOVA was performed on the RTs from the two tasks, with two within-subjects variables of *Condition* and *Task* (Task 1, Task 2). *Condition* interacted with *Task* (*F*_3,69_ = 20.93, *p* < 0.001, *η*^2^ = 0.48).

In Task 1 (i.e., the word-matching task), a significant main effect of *Condition* on the RTs was obtained (*F*_3,69_ = 17.13, *p* < 0.001, *η*^2^ = 0.43); post-hoc comparisons revealed that the RTs in the two cohort conditions were longer than those in the Identical and Unrelated conditions (*ps* < 0.001), indicating larger processing difficulties in the two cohort conditions.

In Task 2 (i.e., the meaning-matching task), a significant main effect of *Condition* on the RTs was also observed (*F*_3,69_ = 64.40, *p* < 0.001, *η*^2^ = 0.74); post-hoc comparisons revealed that, the RTs in the two cohort conditions were longer than those in the Identical and Unrelated conditions (*ps* < 0.01), with no difference between the two cohort conditions. The RTs in the Unrelated condition were also longer than those in the Identical condition (*p* < 0.001), and the response to the Identical condition was the fastest.

Analyses comparing the RTs of the same conditions between the two tasks separately found that the RTs between the two Identical conditions were not different, and the RTs of the other three conditions in Task 2 were longer than those in Task 1 (*ps* < 0.001).

Two-way repeated measures ANOVA was performed on the accuracy data from the two tasks. *Condition* did interact with *Task* as well (*F*_3,69_ = 8.67, *p* < 0.001, *η*^2^ = 0.27).

In Task 1 (word-matching), no significant effect of *Condition* was obtained (*F*_3,69_ = 2.09, *p* = 0.11, *η*^2^ = 0.08).

In Task 2 (meaning-matching), ANOVAs showed a significant effect of *Condition* (*F*_3,69_ = 30.66, *p* < 0.001, *η*^2^ = 0.57). Post-hoc comparisons revealed that response errors in the Synonymous Cohort condition were the largest, and response errors in the Non-synonymous Cohort were also larger than those in the Identical and the Unrelated conditions (*ps* < 0.05), with no difference between the Identical and the Unrelated conditions.

Analyses comparing the accuracy data of the same conditions between the two tasks separately found that the response errors of the Synonymous Cohort in the meaning-matching task were larger than those in the word-matching task (*t*_(23)_ < 2.99, *p* < 0.01).

### ERP results of Group 1 stimuli in Task 1 and Task 2

**Figure 1:**
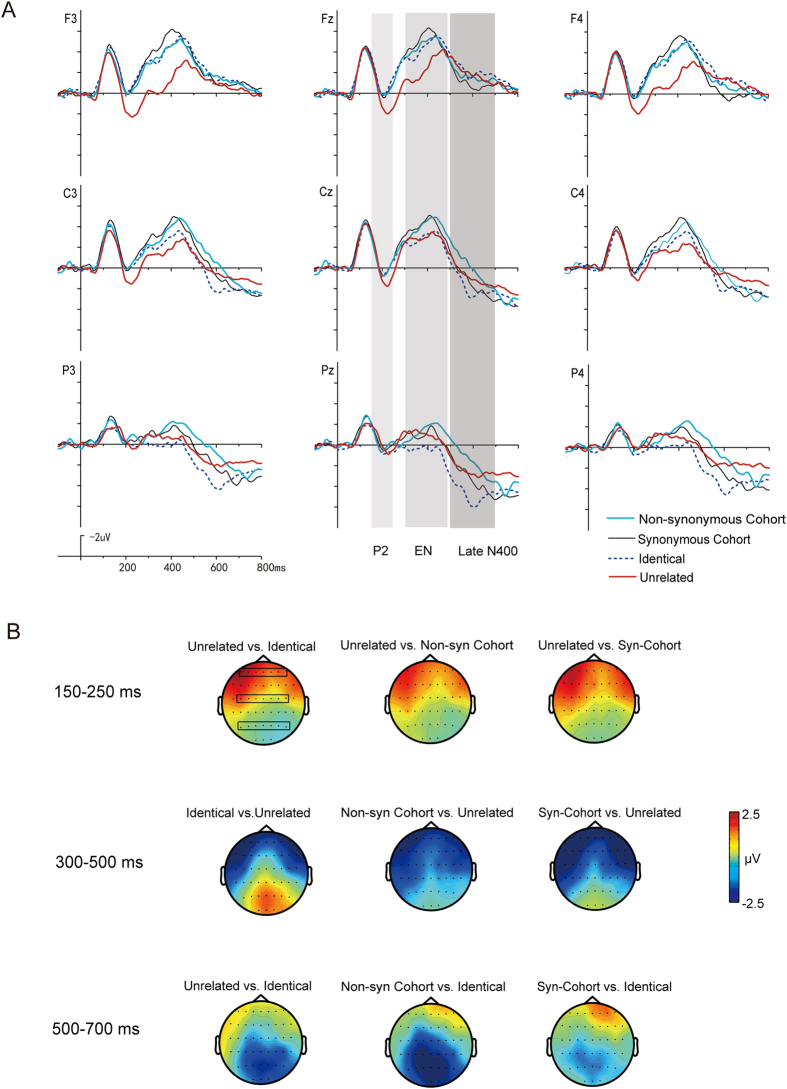
(**A**) Averaged ERP waveforms to Group 1 stimuli in Task 1 (i.e., the word-matching task). (**B**) Topographic distribution of ERP differences of Group 1 stimuli in Task 1.

**Figure 2:**
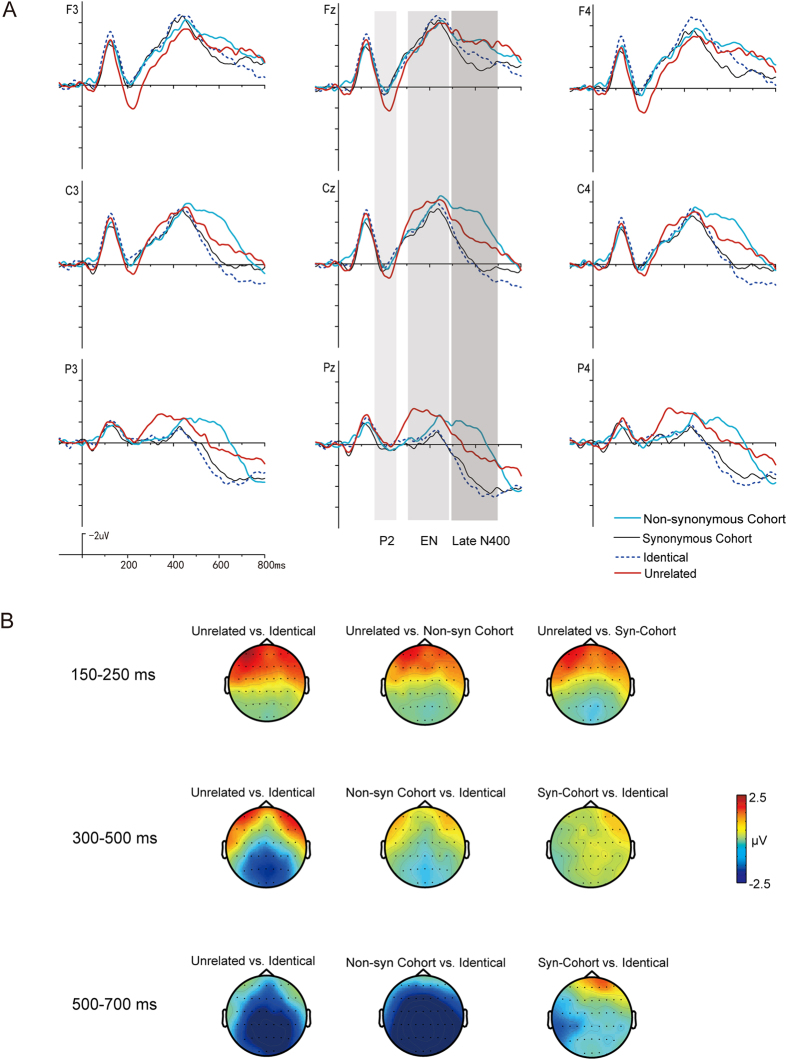
(**A**) Averaged ERP waveforms to Group 1 stimuli in Task 2 (i.e., the meaning-matching task). (**B**) Topographic distribution of ERP differences of Group 1 stimuli in Task 2.

The grand average ERP waveforms for the four conditions of Group 1 stimuli in the two tasks are illustrated in Figs 1 and 2 respectively.

For the P2 (150–250 ms), ANOVAs showed no significant *Task* × *Condition* × *Region* interaction (F_6,138_ = 0.90, p = 0.50, *η*^2^ = 0.04) and no significant *Task* × *Condition* interaction (*F*_3,69_ = 0.64, p = 0.59, *η*^2^ = 0.03). There was a significant interaction between *Condition* and *Region* (*F*_6,138_ = 15.68, *p* < 0.001, *η*^2^ = 0.41). Follow-up analyses comparing the four conditions in both tasks at anterior, central, and posterior sites separately found a significant main effect of *Condition* at anterior sites (*F*_3,69_ = 10.14, *p* < 0.001), with significantly larger positivities elicited by targets in the Unrelated condition than in the other three conditions, while no significant differences were found among these three conditions. At central sites, the main effect of *Condition* was also significant (*F*_3,69_ = 3.92, *p* < 0.05), with larger positivities in the Unrelated condition than the Synonymous Cohort condition. No significant difference among the four conditions was observed at posterior sites (*F*_3,69_ = 0.32, *p* = 0.81).

For the Early N400 (300–500 ms), ANOVAs showed no significant *Task* × *Condition* × *Region* interaction (F_6,138_ = 0.40, p = 0.88, *η*^2^ = 0.02). There was a significant reaction between *Condition* and *Task* (*F*_3,69_ = 11.84, *p* < 0.001, *η*^2^ = 0.34) as well as between *Condition* and *Region* (*F*_6,138_ = 25.67, *p* < 0.001, *η*^2^ = 0.53). Therefore, follow-up analyses were performed within each task separately.

### Early N400 effects for Group 1 stimuli in Task 1 (word-matching)

In Task 1, the *Condition* × *Region* interaction was significant (*F*_6,138_ = 15.03, *p* < 0.001, *η*^2^ = 0.40). At the three regions, the main effects of *Condition* were all significant (*F*_3,69_ = 15.76, *p* < 0.001, *η*^2^ = 0.41; *F*_3,69_ = 9.68, *p* < 0.001, *η*^2^ = 0.30; *F*_3,69_ = 8.32, *p* < 0.001, *η*^2^ = 0.26; respectively). At anterior sites, the Unrelated condition elicited smaller negativities than the other three conditions (*ps* < 0.01), with no difference among the three conditions. At central sites, the two cohort conditions elicited larger negativities than the Unrelated condition (*ps* < 0.01). At posterior sites, the two cohort conditions elicited larger negativities than the Identical condition (*ps* < 0.01), with no difference between the two cohort conditions.

### Early N400 effects for Group 1 stimuli in Task 2 (meaning-matching)

In Task 2, the *Condition* × *Region* interaction was also significant (*F*_6,138_ = 14.55, *p* < 0.001, *η*^2^ = 0.39). Follow-up analyses only found a significant main effect of *Condition* at posterior sites (*F*_3,69_ = 11.33, *p* < 0.001, *η*^2^ = 0.33), with larger negativities elicited in the Unrelated condition than in the Identical and the Synonymous Cohort conditions (*ps* < 0.01).

For the Late N400 (500–700 ms), ANOVAs showed no significant *Task* × *Condition* × *Region* interaction (F_6,138_ = 1.00, p= 0.42, η2 = 0.04). There was a significant interaction between *Condition* and *Task* (*F*_3,69_ = 5.09, *p* < 0.01, *η*^2^ = 0.18) as well as between *Condition* and *Region* (*F*_6,138_ = 14.82, *p* < 0.001, *η*^2^ = 0.39).

### Late N400 effects for Group 1 stimuli in Task 1 (word-matching)

In Task 1, the *Condition* × *Region* interaction was significant for the Late N400 (*F*_6,138_ = 8.98, *p* < 0.001, *η*^2^ = 0.28). At central regions, the main effect of *Condition* was significant (*F*_3,69_ = 3.59, *p* < 0.05, *η*^2^ = 0.13), with larger negativities elicited in the Non-synonymous Cohort condition than in the Identical and the Synonymous Cohort conditions (*ps* < 0.05). At posterior sites, the main effect of *Condition* was significant as well (*F*_3,69_ = 14.50, *p* < 0.001, *η*^2^ = 0.39). The Non-synonymous Cohort and Unrelated conditions were more negative going than the Identical (*ps* < 0.01), and the Synonymous Cohort and the Unrelated conditions were also more negative going than the Identical condition (*ps* < 0.01). Furthermore, the Non-synonymous condition was more negative going than the Synonymous Cohort condition.

### Late N400 effects for Group 1 stimuli in Task 2 (meaning-matching)

In Task 2, the *Condition* × *Region* interaction was also significant (*F*_6,138_ = 10.34, *p* < 0.001, *η*^2^ = 0.31). At anterior sites, the main effect of *Condition* was significant (*F*_3,69_ = 3.72, *p* < 0.05, *η*^2^ = 0.14), with larger negativities elicited in the Non-synonymous Cohort condition than in the Synonymous Cohort conditions (*p* < 0.01). At the central and posterior sites, the main effects of *Condition* were both significant (*F*_3,69_ = 18.85, *p* < 0.001, *η*^2^ = 0.45; *F*_3,69_ = 30.80, *p* < 0.001, *η*^2^ = 0.57, respectively), with more negative going ERPs elicited in the Non-synonymous Cohort and Unrelated conditions than in the Identical and the Synonymous Cohort conditions (*ps* < 0.01).

The aforementioned follow-up analyses of the *Condition* × *Region* interactions on the Early and Late N400 within each task were summarized in Table 2, which showed robust task modulations on both of the components.

### Behavioral results of Group 2 stimuli in Task 2 (meaning-matching)

Reaction time (RT) and accuracy data of Group 2 stimuli are presented in Table 3. Repeated-measures ANOVA for Group 2 stimuli showed a significant main effect of *Condition* on the RTs (*F*_2,46_ = 72.95, *p* < 0.001, *η*^2^ = 0.76). Post-hoc comparisons revealed that, the RTs in the Identical condition were faster than those in the Synonymous and the Unrelated conditions (*ps* < 0.001), with no difference between the Synonymous and the Unrelated conditions. ANOVAs performed on accuracy data showed a significant effect of *Condition* (*F*_2,46_ = 17.94, *p* < 0.001, *η*^2^ = 0.44). Post-hoc comparisons revealed that response accuracy was different in each of the three conditions, with the largest accuracy in the Identical condition and the lowest in the Synonymous condition.

### ERP results of Group 2 stimuli in Task 2 (meaning-matching)

**Figure 3:**
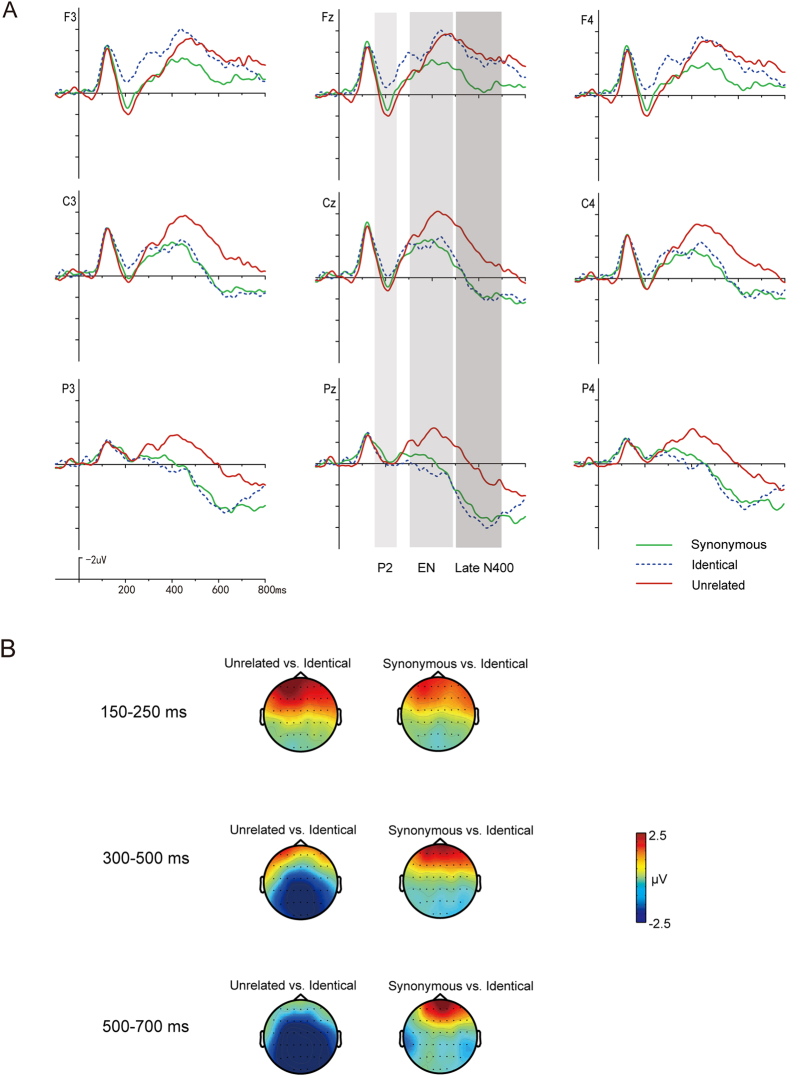
(**A**) Averaged ERP waveforms to Group 2 stimuli in Task 2 (i.e., the meaning-matching task). (**B**) Topographic distribution of ERP differences of Group 2 stimuli in Task 2.

The grand average ERP waveforms to Group 2 stimuli in Task 2 are illustrated in Fig. 3.

For the P2 (150–250 ms), ANOVAs demonstrated a significant interaction effect between *Condition* and *Region* (*F*_4,92_ = 23.47, *p* < 0.001, *η*^2^ = 0.51). Follow-up analyses comparing the three conditions at anterior, central, and posterior sites separately found significant main effects of *Condition* at anterior and central sites (*F*_2,46_ = 20.71, *p* < 0.001, *η*^2^ = 0.47; *F*_2,46_ = 5.23, *p* < 0.01, *η*^2^ = 0.19; respectively), with significantly larger positivities elicited in the Unrelated condition and Synonymous condition than the Identical condition, confirming the phonological P2 effect again.

For the Early N400 (300–500 ms), *Condition* did interact significantly with *Region* as well (*F*_4,92_ = 28.17, *p* < 0.001, *η*^2^ = 0.55). Follow-up analyses comparing the three conditions at anterior, central, and posterior sites separately found that the main effects of *Condition* were all significant (*F*_2,46_ = 8.54, *p* < 0.01, *η*^2^ = 0.27; *F*_2,46_ = 14.29, *p* < 0.001, *η*^2^ = 0.38; *F*_2,46_ = 25.78, *p* < 0.001, *η*^2^ = 0.53, respectively). At anterior sites, the Identical condition elicited larger negativities than the Synonymous condition (*p* < 0.01). At central and posterior sites, the Unrelated condition elicited larger negativities than the Identical and the Synonymous condition (*ps* < 0.01), with no difference between the identical and the Synonymous condition, suggesting a centroparietally distributed semantic effect in the meaning-matching task.

ANOVAs for the Late N400 (500–700 ms) again revealed that *Condition* interacted significantly with *Region* (*F*_4,92_ = 18.06, *p* < 0.001, *η*^2^ = 0.44). Follow-up analyses found the main effects of *Condition* were all significant at the three regions (*F*_2,46_ = 9.38, *p* < 0.001, *η*^2^ = 0.29; *F*_2,46_ = 30.57, *p* < 0.001, *η*^2^ = 0.57; *F*_2,46_ = 41.02, *p* < 0.001, *η*^2^ = 0.64, respectively). At anterior sites, the Unrelated and the Identical conditions elicited larger negativities than the Synonymous condition (*ps* < 0.05), with no difference between the Unrelated and Identical conditions. At central and posterior sites, ERPs to the Unrelated condition were more negative going than the Identical and the Synonymous conditions (*ps* < 0.001), with no difference between the Identical and the Synonymous conditions, again suggesting a centroparietally distributed semantic effect in the meaning-matching task.

### Comparison of condition effects between the two tasks

At the early stage of recognition, P2 effects with similar topographical distribution were elicited by the word-initial phonological mismatches in both tasks, likely indicating the occurrence of similar phonological processing in the two tasks. At the middle stage of recognition, distinguishable early N400 effects with different polarities and topographies were elicited in the two tasks. In Task 1 (word-matching), a fronto-centrally distributed Early N400 effect was elicited by the word-initial phonological similarities, which was more negative than that in the Unrelated condition. Whereas, a parietally distributed N400 (as the typical semantic N400) was elicited by the Unrelated conditions in Task 2 (meaning-matching). At the late stage of recognition, similar centroparietal Late N400 effects were elicited in both tasks.

ERP differences (in *μ*V) between the Identical and the other three conditions of Group 1 stimuli within each task were given in Table 4.

In order to compare the between-task differences in the condition effects (i.e., ERP amplitude difference between the Identical and one of the other three conditions of Group 1 stimuli) summarized in Table 4, three-way repeated measures ANOVA was performed on the P2 effect and the Late N400 effect separately, with three within-subjects variables of *Task*, *Condition Effect* (Non-synonymous Cohort, Synonymous Cohort, Unrelated), and *Region* (anterior, central, posterior). For the P2, ANOVA showed no significant effects involving *Task*, indicating that the phonological P2 effect is not sensitive to the difference of the tasks in this study. For the Late N400 effect, there was a significant interaction between *Condition Effect* and *Task* (*F*_2,46_ = 5.28, *p* < 0.01, *η*^2^ = 0.19). Follow-up analyses comparing the condition effect of one same condition between two tasks found that the Non-synonymous conditions between the two tasks were significantly different with respect to the Late N400 (*F*_1,23_ = 10.14, *p* < 0.01), with larger condition effects in the meaning-matching task (Task 2, −3.76 and −4.2 *μ*V) than in the word-matching task (Task 1, −1.36 and −2.44 *μ*V) (Table 3). The condition effect of the Unrelated conditions trended towards significant difference between the two tasks (Task 1: −0.7 and −1.8 *μ*V, Task 2: −2.11 and −2.83 *μ*V; *F*_1,23_ = 3.45, *p* = 0.07). These larger condition effects for the same conditions in Task 2 may indicate a larger demand of semantic processing in the meaning-matching task.

## Discussion

In the present study, the word-matching task and the meaning-matching task were administrated in one experiment to investigate how the phonological processing and the semantic processing in spoken word recognition are modulated by task requirements. The RTs of the two cohort conditions in both tasks were the longest among all conditions, indicating an inhibitory effect of the word-initial phonological similarities on spoken word recognition. Similar phonological mismatch-associated P2 effect, which is independent of the semantic similarities between the primes and the targets, was elicited in both tasks. Divergences between the two tasks were observed mainly at the middle and late stages of recognition. In the time window of 300–500 ms post-stimulus onset, a frontal Early N400 (phonological N400) reflecting the phonological disambiguating process, was elicited in the word-matching task. However, a parietally distributed Early N400 component (semantic N400) indicating the initiation of the semantic processing, was elicited in the meaning-matching task. In the time window of 500–700 ms, the centroparietal Late N400 components were elicited in both tasks, but with larger effects in the meaning-matching task.

Both tasks in the current study replicated the phonological mismatch-associated P2 effect (i.e., a larger fronto-centrally distributed P2 in the 150–250 ms time window elicited by word-initial mismatches) demonstrated by Huang and colleagues^17^. The Non-synonymous Cohort and Synonymous Cohort conditions of Group 1 stimuli elicited a similar P2 component, so did the Synonymous condition and the Unrelated condition of Group 2, suggesting that the phonological P2 effect was not modulated by the semantic relations between the primes and targets.

P2 was not observed in cross-modal studies^8^,^9^ or studies using sentence as context^2^,^5^,^6^. The specific paradigm employed probably accounts for the more prominent phonological mismatch effects on the P2 rather than the PMN in the current study. Specifically, a cross-modal task may not activate the most typically used spoken word processing routes^16^. Compared to a cross modality design, there would be more phonological links between the prime and target when both were presented through the auditory channel, as Gaskell and Marslen-Wilson have pointed out^18^. While in many unimodal auditory tasks, such as shadowing^19^, phoneme deletion^20^, or combination of vowel and consonant-vowel^21^, participants were asked to rebuild or generate the representations of the targets on the basis of the prime stimuli, so the targets were not physically presented in these studies. The processing system was thus given less opportunity to benefit directly from the primes. In this study, it is reasonable to argue that the activated phonological representations of the primes are temporarily available in the phonological buffer of working memory^22^,^23^,^24^ and reduce the phonological activation demand for target words in the related conditions. Thus, the P2 mismatch effect found in the present study appears to reflect the facilitatory effect of the directly activated similar phonological representation at the pre-lexical (phonological) processing stage (i.e., reducing phonological processing load). Similar P2 effects were elicited in both tasks, indicating that the P2 is a robust index of phonological processing at the pre-lexical level during spoken word recognition. More other experimental tasks and paradigms are recommended to further examine whether the phonological processing at this stage is task-irrelevant, automatic and not modulated by the top-down mechanism.

As indicated by the results of this study, in the 300–500 ms time window, Early N400 effects with different polarities and topographies were elicited in the two tasks, indicating different processing occurred between the two tasks. In Task 1 (the word-matching task), a fronto-central negative component was elicited in conditions with word-initial phonological similarities between 300 and 500 ms post-target onset, which again replicated Huang and colleagues^17^. It should be noted that the Early N400 appears to be a component different from the PMN, since its amplitude was modulated by phonological mismatch in an opposite direction compared to the PMN. The topographic distribution of the Early N400 in Task 1 is different from the typical larger semantic N400 to the unrelated condition in literature^2^,^8^,^9^,^10^,^11^,^12^. Similar amplitudes of the Early N400 were elicited as a result of the overlapped phonemes with the target words in the two cohort and identical conditions, suggesting that this Early N400 effect reflects disambiguation/selection amongst multiple phonological candidates activated by the repeated word-initial phonemes in these conditions.

In Task 2 (the meaning-matching task), the Unrelated conditions in both word groups elicited larger negativities than the other conditions at the central and posterior regions. The Synonymous Cohort of Group 1 stimuli, which shared similar word-initial syllables and similar meanings with the primes, elicited an Early N400 with similar amplitudes as the Identical condition. And the synonymous targets of Group 2 stimuli, which shared no phonological similarity with the primes, elicited a similar Early N400 component as the Identical condition. These results suggested that the Early N400 in Task 2 is primarily sensitive to the semantic attributes of the speech input. This component showed a similar topographic distribution as the classic semantic N400^8^,^9^,^10^,^11^,^12^, indicating that the semantic processes in the meaning-matching task are initiated in this time interval.

As reflected by the different results of the two tasks in this study, the effects of the phonological and semantic processing at the middle stage are modulated by the top-down mechanisms. It is consistent with the prior studies that the phonological processing of the rhymes is, to some degree, task-relevant and modulated by top-down mechanisms^14^,^15^,^25^.

At the late stage, a centro-parietally distributed Late N400 was elicited in both tasks. However, this effect was larger in the meaning-matching task (i.e., Task 2) than in the word-matching task (i.e., Task 1), indicating deeper semantic processing in Task 2. The Non-synonymous Cohort condition, i.e., the first syllables of the primes and the targets were the same but their meanings were unrelated, elicited the largest Late N400 in both tasks, indicating that phonological manipulations also have some effects on the Late N400. These ERP results are consistent with those of the cohort condition in prior studies^2^,^8^,^9^,^17^. In both tasks, the Identical conditions of both word groups elicited the smallest Late N400 as expected. And there were more prominent positivities after 500 ms to the repeated words in the two tasks and also to synonymous words in Task 2, which are consistent with the well-documented late positive component (LPC) repetition effect associated with episodic/declarative memory^26^. Though the same/very close meanings were shared by the primes and the targets, the Synonymous Cohort elicited larger Late N400 than the Identical condition at the posterior sites in Task 1. However, the Synonymous Cohort elicited nearly similar Late N400 as the Identical condition in Task 2. Such difference confirms that the word-matching paradigm is shallower in processing, and emphasizes the phonological matching/discriminating process.

Importantly, the Synonymous Cohort in Task 1 elicited smaller Late N400 than the Non-synonymous Cohort at the centroparietal sites, suggesting that the semantic processing of the targets was relatively automatic. The attenuation of the Late N400 in the Synonymous Cohort condition likely results from the semantic activation associated with processing of the primes that automatically spreads through the semantic network towards representation of the target words, even in the more phonologically oriented word-matching task. This result is consistent with studies using active and passive paradigms^13^, and studies using selective attention paradigms^14^,^15^. The result is also consistent a functional MRI (fMRI) study^27^ in which the semantic activation occurred even when the subjects’ attention was directed to nonverbal properties of the speech input such as the speakers’ voice.

The distinguishable condition effects on the Late N400 component between the two tasks suggest that the semantic activation of the target words is relatively automatic, but the magnitude of their semantic activation can be modulated by task requirements. This is in line with an early study of Holcomb^28^, in which the N400 was larger when participants were instructed to attend than to ignore the semantic relationships of the prime-target pairs in a lexical decision task. This result of the present study is also consistent with a study of Hohlfeld and Sommer^29^, in which the semantic activation of the spoken words can be attenuated by additional task load. These results together indicate that the degree of the semantic activation at late stage of spoken word recognition can also be modulated by top-down processes.

The N400 effect found in the meaning-matching task of this study is consistent with the results of Liu and colleagues^6^, in which an enhancement of N400 was elicited by the incongruous terminal disyllabic words in sentence context. And the N400 effects found in this study are different from the N400 effects in prior studies on Chinese monosyllabic spoken word recognition^9^,^30^,^31^,^32^, in which the N400 effects can reflect the tonal and segmental processing of the monosyllabic words. Possible reasons for the discrepancies are the different materials and paradigms employed. The monosyllabic words in Mandarin Chinese are much simpler than disyllabic words, likely leading to different recognition mechanisms to these two types of words. For example, a set of word candidates will be activated simultaneously when the first syllables of the disyllabic words are perceived, and these multiple candidate words will compete among each other^33^,^34^,^35^. Therefore, more processing demands of word disambiguation/selection amongst multiple phonological candidates will be needed during disyllabic words access. These processing difference between disyllabic words and monosyllabic words are evident in the two experiments of Huang and colleagues^17^. More studies on monosyllabic and disyllabic spoken word recognition in Chinese and in the other languages are warranted.

Taken together, by using unimodal tasks of word-matching and meaning-matching, this study investigated how the phonological processing and semantic processing in spoken word recognition are modulated by top-down mechanism induced by task requirements. From the results of this study, the time course of Chinese disyllabic spoken word recognition can be divided into three sub-stages. In the early stage (i.e., ~150–250 ms post-stimulus onset), phonological processing indexed by the P2 effect seems not sensitive to the difference task requirements involved in the current study. No significant modulation of semantic similarities between the primes and targets on the P2 lends further support for the existence of an early phonological processing stage prior to semantic processing during spoken word recognition. While in the middle stage (i.e., ~300–500 ms), the processes are modulated by task requirements, and both the semantic and phonological processes can be involved in this time range. In the late stage (i.e., ~500–700 ms), the semantic representation of the spoken words can be activated automatically, but the magnitude of the semantic activation is task-dependent. Thus, our results fit with a partially-serial and sequential model for spoken word recognition, and indicate that spoken language processing is a dynamic and flexible system from very fundamental levels. Given the significant difference between Mandarin Chinese and Indo-European languages, more studies using the unimodal paradigms as employed in the present study are recommended to examine which effects documented here are language-universal and which are specific to disyllabic spoken word recognition in Mandarin Chinese.

## Methods

### Participants

The study was conducted in accordance with the ethical standards prescribed by the Ethics Committee of Chinese Psychological Society. All the experimental protocols were approved by the Capital Normal University Review Board. All individuals provided written informed consent prior to participation in the study. Twenty-four right-handed undergraduate and graduate students (10 males, 14 females, mean age = 21.8, age range = 19–26) from the Capital Normal University in Beijing participated in the experiment. They were all native Mandarin Chinese speakers, and reported neither speech/hearing problems, nor neurological disorders. Subjects were paid for their participation.

### Materials

Disyllabic materials were used as stimuli in this study. These words were selected from the Sixth edition of the Contemporary Chinese Dictionary^36^. According to the phonological and semantic relations within each word pair, the stimuli were divided into two groups. In the first group (Group 1), both phonological similarities and semantic similarities were shared by the prime and the target, and one same target preceded by different primes in four conditions. In the second group (Group 2), only the semantic similarities were shared within each word pair, with no phonological similarities involved. Again, one same target word was primed by three different types of primes. The example stimuli for each condition were given in Table 5.

There were 90 target words of Group 1, with half used in each task (i.e., the word-matching task and the meaning-matching task). There were 45 target words of Group 2, which were only used in Task 2 (i.e., meaning-matching). In both tasks, there were fillers to balance the frequency of correct responses. 180 test trials and 90 filler trials constructed in Task 1 were split into 4 blocks. 315 test trials and 45 filler trials in Task 2 were split into 5 blocks. Auditory words were read by a male radio broadcaster at normal pace. The sounds were digitally recorded at 16 bits with a sampling rate of 44,100 Hz, edited by Praat 5.1.20. (http://www.fon.hum.uva.nl/praat/download_win.html). The mean duration was 279 ms (SD = 34) for the first syllable of the targets, and 298 ms (SD = 47) for the second syllables.

### Procedure

Participants were individually tested in a dimly lit, acoustically and electrically shielded room. Presentation 0.71 (Neurobehavioral System, Albany, California, USA) was used for stimulus presentation and behavioral responses collecting. The word-matching and meaning-matching tasks were given in two task sessions, with the order of the two tasks counter-balanced across subjects. Appropriate task instructions were given right before each session, and practice was performed to make subjects become familiar with the tasks. At the beginning of each trial, a fixation cross appeared for 300~600 ms (jittered, mean = 450 ms) on a PC monitor, then a pair of prime and target were played, with a 1500~2000 ms jittered stimulus onset asynchrony (SOA, mean = 1750 ms). The fixation cross remained on the screen for 3500 ms after the prime onset, followed by a blank screen of 1000~1500 ms (jittered, mean = 1250 ms) before the next trial. Auditory word stimuli were presented at a comfortable level through a pair of headphones (HUAWEI AM115).

The task types served as a within-subject factor. When hearing the second word in each pair, subjects were asked to judge whether the two words were identical in the word-matching task (Task 1), and whether the meaning of the two words were the same in the meaning-matching task (Task 2), by pressing the left or the right button on the mouse with two thumbs fast and accurately. Half of the subjects fulfilled Task 1 first, and the other half performed Task 2 first. The assignment of the left/right buttons to the yes/no responses was counterbalanced across subjects. Participants were instructed to fixate the cross at the center of the screen while minimizing head movement and eye blinks during the experiment. The order of the stimulus presentation was pseudo-randomized, with the very first two trials in each block always being fillers. The entire experiment lasted for about 60 minutes.

### ERP recording and data analysis

Electroencephalography (EEG) was recorded using a 64-channel Quik-cap (Neuroscan Inc., El Paso, Texas, USA) with electrodes positioned according to the international 10–20 system. The scalp-electrode impedances were kept below 5 KΩ. The EEG signal was sampled at a rate of 500 Hz. All electrodes were referenced to the left mastoid during online recording and re-referenced offline to the averaged mastoids. Bipolar vertical and horizontal electrooculography (EOG) were also measured. The continuous EEG data were segmented into epochs of −100 to 800 ms relative to the onset of the second spoken words (i.e., targets) within each trial. Data were then filtered offline using a 24 dB zero-phase-shift digital bandpass filter (0.05–30 Hz). Epochs were baseline corrected with the average voltage of the pre-stimulus interval. EEG to trials with incorrect behavioral responses were excluded from data analysis. Trials containing eye blinks and other excessive artifacts were also rejected using a maximum-voltage criterion of ±60 *μ*V at all scalp electrodes. There were over 37 clean epochs included into averaging for each condition in Task 1, and over 35 clean epochs included into averaging for each condition in Task 2.

Mean amplitudes were measured for three ERP components: the P2 (150–250 ms), the Early N400 (300–500 ms), and the Late N400 (500–700 ms). For Group 1 stimuli, three-way repeated measures analysis of variance (ANOVA) was performed on the mean amplitude of each component, with three within-subjects variable of *Task*, *Condition*, and *Region* (anterior, central, posterior). For Group 2 stimuli, two-way repeated measures ANOVA was performed on the mean amplitude of each component, with two within-subjects variable of *Condition* and *Region* (anterior, central, posterior). For the factors *Region*, 21 selected electrodes were divided into 3 clusters: anterior (F5, F3, F1, Fz, F2, F4, F6); central (C5, C3, C1, Cz, C2, C4, C6); posterior (P5, P3, P1, Pz, P2, P4, P6). Only significant effects were reported. Greenhouse-Geisser correction (Greenhouse & Geisser, 1959) was applied to correct for violations of the sphercity assumption when appropriate. Whenever applicable, Bonforroni correction was used to counteract the problem of multiple comparisons.

## Additional Information

**How to cite this article**: Huang, X. *et al.* Task modulation of disyllabic spoken word recognition in Mandarin Chinese: a unimodal ERP study. *Sci. Rep.* **6**, 25916; doi: 10.1038/srep25916 (2016).

## Figures and Tables

**Table 1:** Mean RT and accuracy for each condition (Mean (SD)) of Group 1 stimuli in the two tasks.

Task	Condition	RT(ms)	ACC(%)
Task 1	Non-synonymous Cohort	794 (109)	97.8 (3.7)
	Synonymous Cohort	796 (114)	96.0 (5.9)
	Identical	740 (100)	97.7 (2.8)
	Unrelated	749 (138)	98.0 (3.4)
Task 2	Non-synonymous Cohort	921 (109)	96.1 (5.0)
	Synonymous Cohort	893 (113)	91.0 (5.6)
	Identical	752 (97)	98.4 (2.8)
	Unrelated	846 (127)	99.0 (2.0)

**Table 2:** Follow-up analyses of the *Condition* × *Region* interactions on the Early and Late N400 for Group 1 stimuli.

Effect	Region	In Task 1	In Task 2
EN	Anterior	(N.S. among Non-syn Cohort, Syn Cohort, and Identical) < Unrelated	N.S.
	Central	(N.S. between Non-syn Cohort and Syn Cohort) < Unrelated Non-syn Cohort < Identical	N.S.
	Posterior	(N.S. between Non-syn Cohort and Syn Cohort) < Identical	Unrelated < (N.S. between Syn-Cohort and Identical)
LN	Anterior	N.S.	Non-syn Cohort < Syn Cohort
	Central	Non-syn Cohort < (N.S. between Syn Cohort and Identical)	Unrelated < Non-syn Cohort < (N.S. between Syn Cohort and Identical)
	Posterior	(N.S. between Non-syn Cohort and Unrelated, N.S. between Syn Cohort and Unrelated) < Identical Non-syn Cohort < Syn Cohort	(N.S. between Unrelated and Non-syn Cohort ) < (N.S. between Syn Cohort and Identical)

**Table 3:** Mean RT and accuracy for each condition (Mean (SD)) of Group 2 stimuli in Task 2.

Target Group	Condition	RT(ms)	ACC(%)
Group 2	Synonymous	838 (119)	94.6 (4.4)
	Identical	731 (104)	99.4 (1.0)
	Unrelated	847 (119)	98.0 (2.7)

**Table 4:** ERP differences (in *μ*V) between the Identical and the other three conditions of Group 1 stimuli in each task.

Effect	Contrast	Region	In Task 1	In Task 2
P2	Non-synonymous Cohort vs. Identical	Anterior	0.36	0.25
		Central	0.05	0.41
		Posterior	−0.31	0.26
	Synonymous Cohort vs. Identical	Anterior	0.15	0.28
		Central	−0.17	0.48
		Posterior	−0.38	0.50
	Unrelated vs. Identical	Anterior	1.68**	1.79**
		Central	0.80 (p = 0.06)	0.99*
		Posterior	−0.29	−0.02
EN	Non-synonymous Cohort vs. Identical	Anterior	0.08	0.62
		Central	−0.86*	−0.05
		Posterior	−1.73**	−0.67
	Synonymous Cohort vs. Identical	Anterior	−0.54	0.44
		Central	−1.04*	0.34
		Posterior	−1.36*	0.21
	Unrelated vs. Identical	Anterior	2.37**	1.24*
		Central	0.77	−0.40
		Posterior	−0.89	−1.80**
LN	Non-synonymous Cohort vs. Identical	Anterior	0.40	−1.04
		Central	−1.36**	−3.76***
		Posterior	−2.44***	−4.20***
	Synonymous Cohort vs. Identical	Anterior	0.88 (p = 0.05)	0.75
		Central	−0.39	−0.61
		Posterior	−1.14*	−0.72
	Unrelated vs. Identical	Anterior	0.37	−0.62
		Central	−0.70	−2.11**
		Posterior	−1.80**	−2.83***

^*^P < 0.05 (two-tailed).

^**^P < 0.01 (two-tailed).

^***^P < 0.001 (two-tailed).

**Table 5:** Example stimuli and expected responses for each condition.

Stimuli	Condition	Prime-Target	Expected Response
Group 1 in Task 1	Non-synonymous Cohort, i.e., the first syllables of the prime and target were the same but the meaning of the two words was unrelated.	zao3tui4—zao3fan4 (“leave early”—“ breakfast”)	N
	Synonymous Cohort, i.e., the first syllables of the prime and target were the same and the two words were synonymous.	zao3can1—zao3fan4 (both mean “breakfast”)	N
	Identical, i.e., the prime and the target were the same words.	zao3fan4—zao3fan4 (“breakfast”—“ breakfast”)	Y
	Unrelated, i.e., the two words were not related either phonologically or semantically.	shou3duan4—zao3fan4 (“means”—“ breakfast”)	N
Group 1 in Task 2	Non-synonymous Cohort	gong1yu4—gong1zheng4 (“apartment”—“justice”)	N
	Synonymous Cohort	gong1ping2—gong1zheng4 (both mean “justice”)	Y
	Identical	gong1zheng4—gong1zheng4 (“justice”—“justice”)	Y
	Unrelated	ran2hou4—gong1zheng4 (“then”—“justice”)	N
Group 2 in Task 2	Synonymous	gong1zi1—xin1shui3 (“both mean salary”)	Y
	Identical	xin1shui3—xin1shui3 (“salary”—“salary”)	Y
	Unrelated	gai4nian4—xin1shui3 (“concept”—“salary”)	N
